# What Do I Do if the Valve Don't Pass? Use a Snare Catheter!

**DOI:** 10.1002/ccr3.9628

**Published:** 2024-12-19

**Authors:** Simon Berger, Nicolas Combaret, Geraud Souteyrand

**Affiliations:** ^1^ Department of Cardiology Clermont‐Ferrand University Hospital Center Clermont‐Ferrand France; ^2^ Department of Cardiology Clermont‐Ferrand University Hospital Center, CNRS, Clermont Auvergne University Clermont‐Ferrand France

**Keywords:** calcification, severe aortic stenosis, snare technique, transcutaneous aortic valve replacement, valve replacement

## Abstract

We report a case of a complex transcatheter aortic valve implantation (TAVI) complicated by severe calcifications, which prevented the delivery system from advancing through the aortic valve. To address this challenge, we employed an innovative solution using a Snare catheter. This approach enabled stabilization and guidance of the delivery system, facilitating the crossing of the calcified obstruction and the successful completion of the procedure. This case highlights the utility of the Snare catheter as a complementary technique to overcome anatomical challenges in complex TAVI procedures.

**Abbreviations**
SC: snare catheter
TAVI: transcatheter aortic valve implantation
THV: transcatheter heart valve

**Summary**

- The use of snare catheter could change the orientation and angulation of the TAVI delivery system and permit crossing of heavy calcified aortic valves without complication.

## 1 Introduction

The Sapien transcatheter heart valve (THV) (EDWARDS Lifesciences) is currently one of the most widely used devices for transcatheter aortic valve implantation (TAVI) worldwide.

The Edwards THV device is distinguished by its torque mechanism and typically navigates aortic stenosis effortlessly and without the need for predilation.

We report a case of aortic stenosis where the THV encountered unexpected crossing difficulties, not predicted by CT scan analysis. Despite performing predilation, positioning THV prosthesis was unfeasible due to unfavorable calcification pattern. The deployment of the Sapien valve was successful thanks to the use of a snare catheter which facilitated crossing the native valve.

## 2 Case History

An 88‐year‐old patient was admitted to our hospital after experiencing his first episode of cardiac failure and was diagnosed with a severe aortic stenosis.

### 2.1 Past Medical History

The patient's past cardiovascular medical history revealed multiple coronary lesions treated by percutaneous coronary intervention (PCI): the first diagonal in 1995 and the proximal left anterior descending artery, the ostial right coronary artery and the left circumflex in 2015. A permanent pacemaker was implanted in 2020, after an episode of high‐grade atrioventricular (AV) block, currently in atrial fibrillation for which he is anticoagulated with Eliquis.

### 2.2 Investigations

**FIGURE 1:**
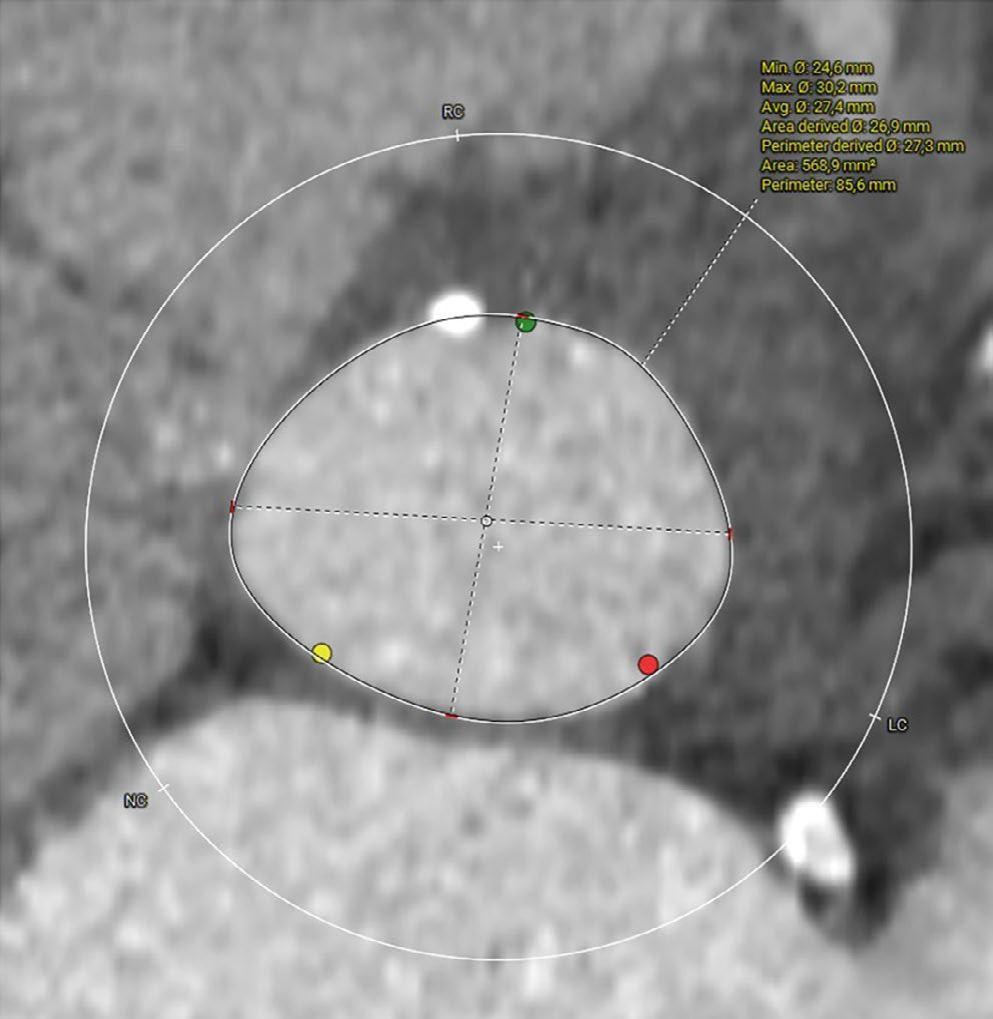
Measurement of the aortic ring size and prosthesis selection based on the pre‐TAVI CT scan.

**FIGURE 2:**
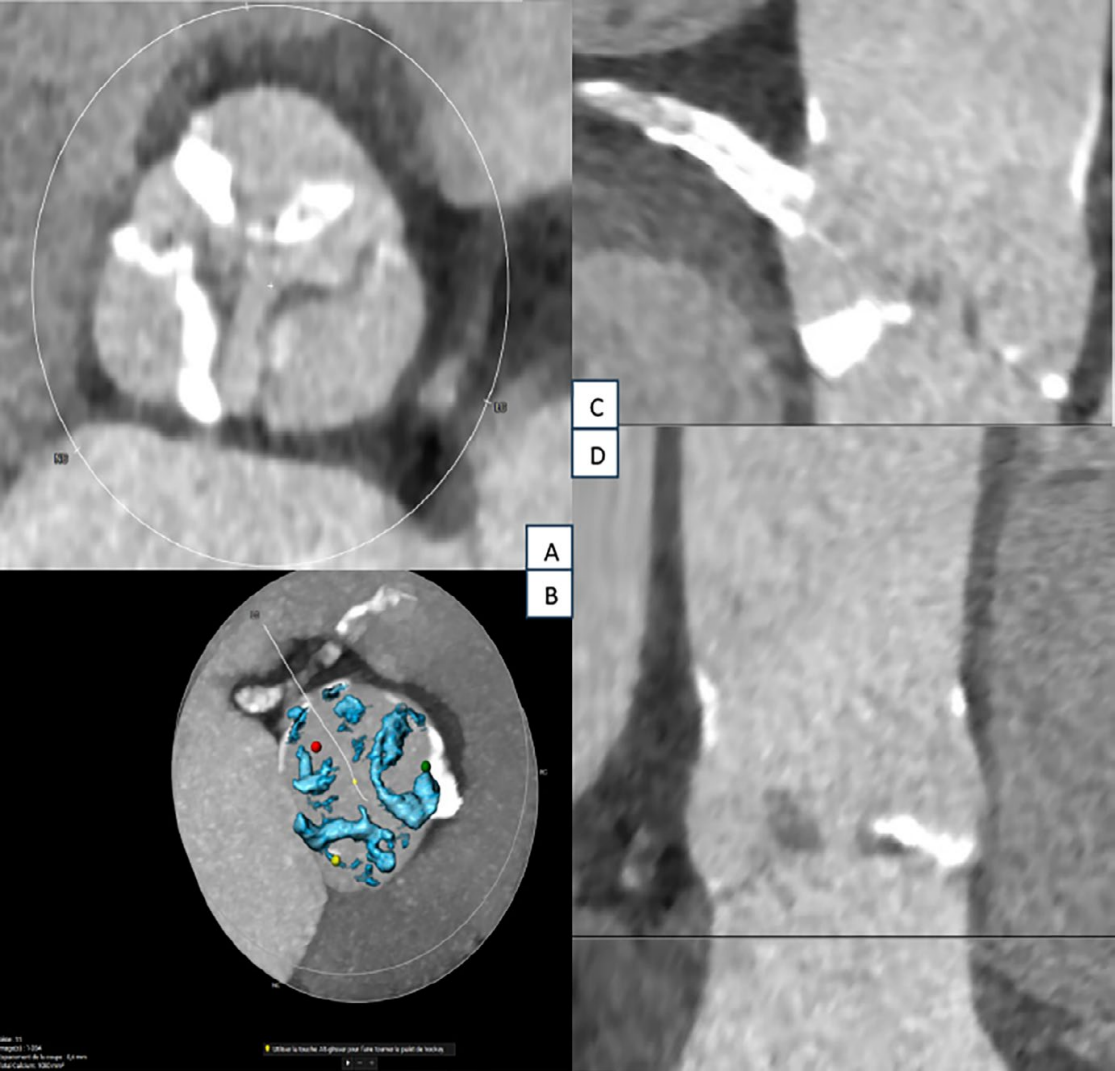
Reconstruction of the aortic valve using pre‐TAVI CT scan to highligth calcifications in the left coronary and non‐coronary cusps. (A) Illustration showing calcifications in the left coronary and non‐coronary cusps. (B) 3D reconstruction of the cusps with calcification score. (C and D) Coronal sections depicting calcifications in the left coronary and non‐coronary cusps.

**FIGURE 3:**
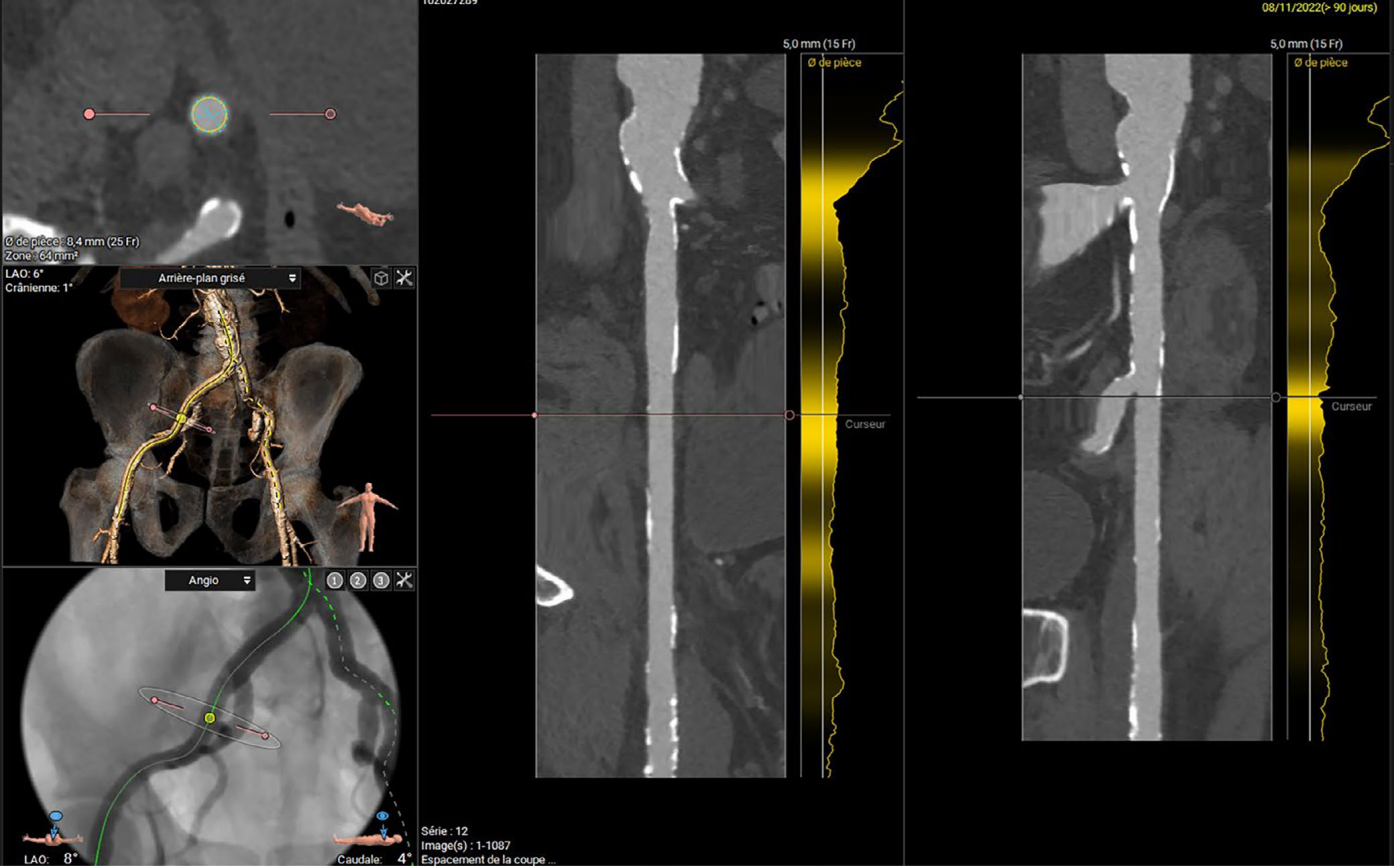
3D reconstruction of vascular axes illustrating femoral artery selection and calcifications of the descending aorta (sagittal cross‐section).

The pre‐procedural assessment included:

1. Transthoracic echocardiography showed a concentric left ventricular hypertrophy and a moderate alteration of the left ventricular ejection fraction, estimated at 45%. It also revealed an infero‐septo‐apical infarct scar. The mean transvalvular aortic gradient was estimated at 35 mmHg, the maximal transvalvular speed at 3.7 cm/s, and the aortic valve area at 0.84 cm^2^. The systolic function of the right ventricle was preserved, with moderate pulmonary hypertension evaluated at 50 mmHg.
2. Coronary angiography revealed a significant intra‐stent restenosis in the proximal right coronary artery, treated with an OPTIMAX 4.5 × 16 mm bare metal stent.
3. Pre‐TAVI CT scan showed unobstructed ilio femoral axes, enabling the selection of the right transfemoral approach for the procedure. It evaluated the native aortic annulus size at 26 mm, with a perimeter of 27.3 mm and an area of 26.9 mm (Figure 1). Notably, CT imaging revealed calcification on at least 2 leaflets of the aortic valve, with a calcification score of 1080 mm^3^, suggesting a non‐complex procedure (Figures 1, 2, 3). The patient underwent geriatric assessment and anesthesia consultation prior to the procedure.

## 3 Methods

The procedure was performed under local anesthesia using a right transfemoral access, with right transradial secondary access to guide the valve implantation. The right femoral puncture was executed under ultrasound guidance.

The Edwards eSheath+ introducer set introduced on a Safari guidewire (Boston Scientific) without encountering any difficulties. Subsequently, the Safari2 pre‐shaped small guidewire was inserted into the left ventricle.

**FIGURE 4:**
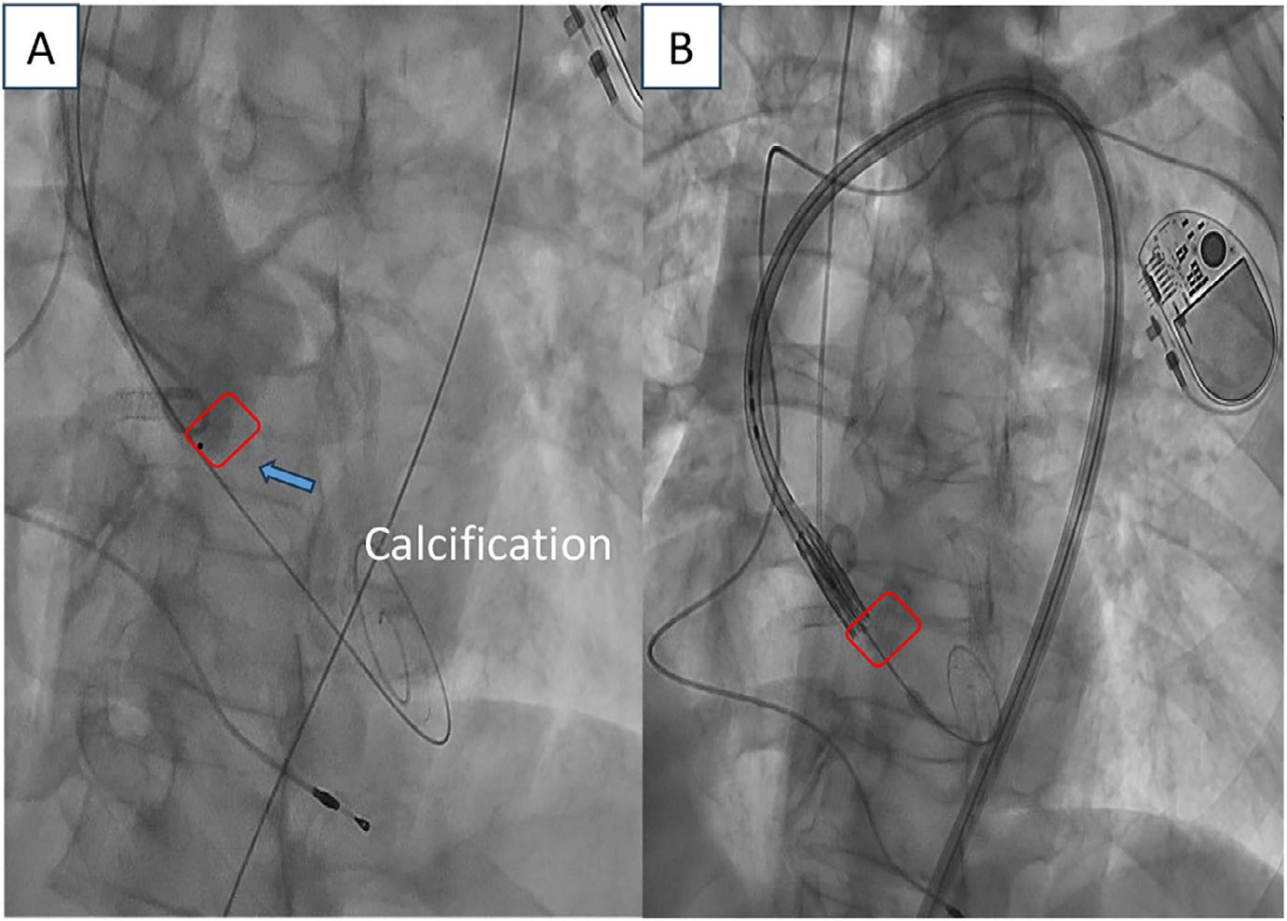
Fluoroscopic images taken during the unsuccessful positioning of the delivery system. (A) Blue arrow indicating calcification in the non‐coronary cusp. (B) Failure of the Edwards THV device to cross the native aortic stenosis location.

However, when introducing the Edwards SAPIEN 3 ULTRA 26 mm valve delivery system to the aortic valve, it failed to navigate through the native aortic stenosis. In an attempt to facilitate crossing, the distal part of the Edwards balloon was inflated, but this did not aid in overcoming the obstruction. The patient experienced pain upon encountering an unfavorable calcification pattern on the native valve. Despite numerous attempts and manipulations with the torque device, the crossing remained unsuccessful (Figure 4). Consequently, a left transfemoral arterial approach was performed, with a new attempt to navigate the native aortic valve using an Amplatz Ultra Stiff 0.035 × 260 cm guidewire, aiming to create a rail effect with two rigid guidewires. Nevertheless, this strategy was also unable to facilitate crossing the native valve.

By the right femoral access, a predilatation using a 20 mm VACSII balloon (OPSENS) was performed but did not facilitate crossing the native aortic valve.

**FIGURE 5:**
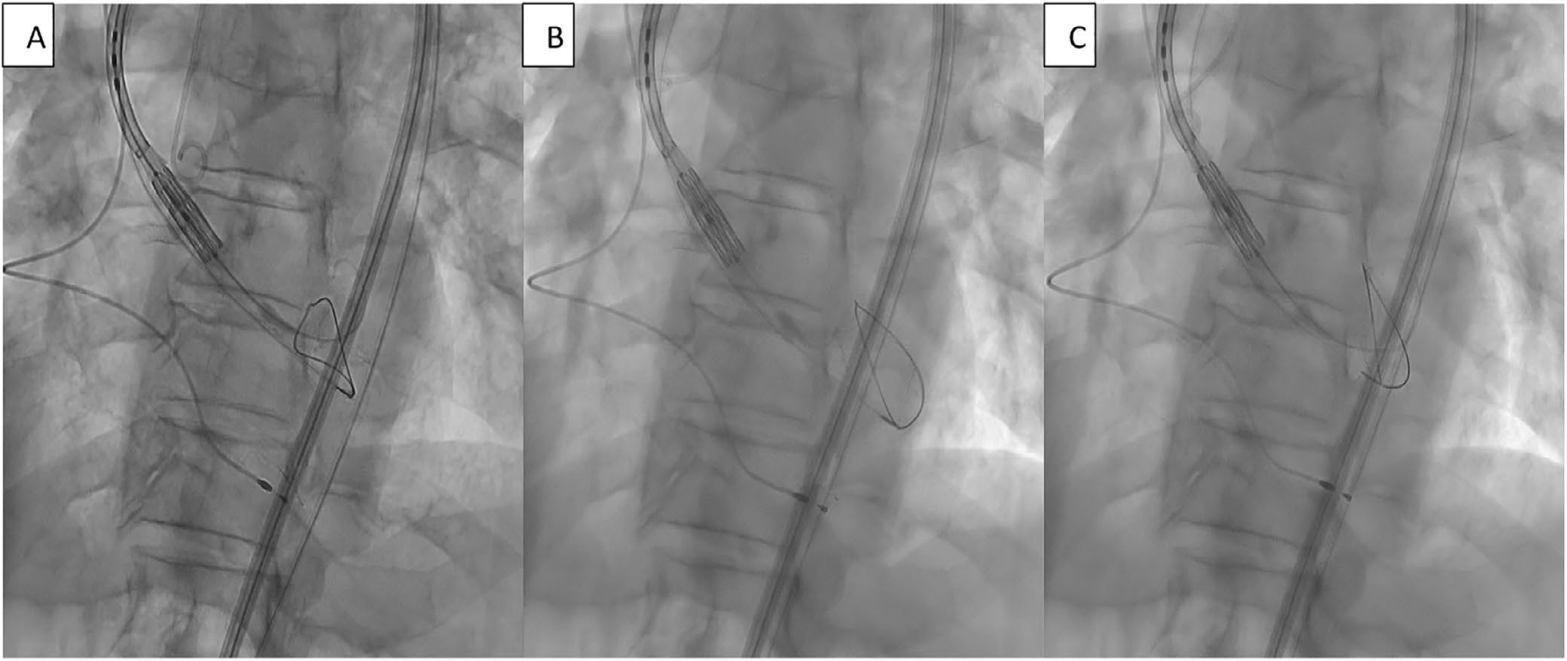
Fluoroscopic images captured during the setup of the Snare Catheter. (A) Insertion of the Snare Catheter (SC) via the left femoral artery into the left ventricle. (B) Withdrawal of the SC to the tip of the delivery system. (C) Advancement of the Safari 2 guide through the loop of the SC, enablng further advancement of the SC to the level of the delivery system.

**FIGURE 6:**
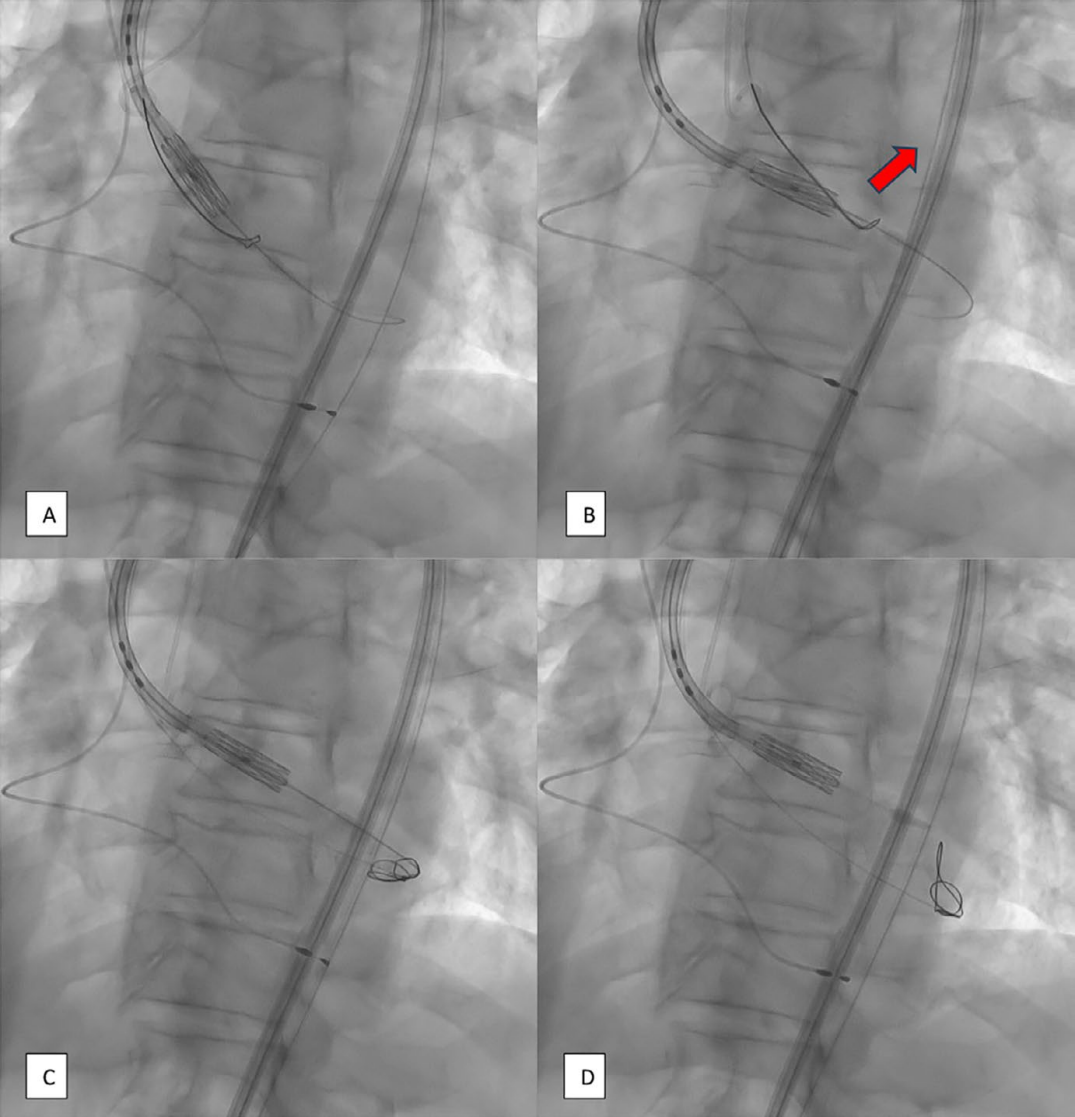
Fluoroscopic images showcasing the positioning of the delivery system and the removal of the Snare Catheter. (A) SC progresses to the THV delivery device. (B) Application of SC traction to achieve the correct angulation for valve crossing. (C) SC is pushed to the bottom of the left ventricle. (D) Removal of the Safari 2 guidewire, facilitating the release and extraction of the SC.

**FIGURE 7:**
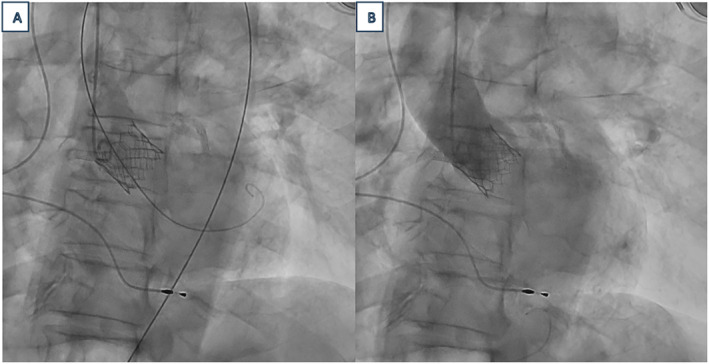
Fluoroscopic images demonstrating successful delivery of the device through the aortic valve without (A) and with (B) the use of a contrast agent (B).

Subsequently, we employed an Amplatz Goose Neck Micro‐lasso 2 × 175 cm snare catheter to attempt repositioning of the prosthesis. The snare catheter (SC) was introduced via the left transfemoral artery and advanced to the left ventricle. Despite multiple attempts to position the Safari 2 guidewire in the snare loop, we were unsuccessful. Finally, we removed the guide wire from the nose cone of the delivery catheter, positioned the loop of the snare in front of the nose cone and pushed the guidewire through the loop. (Figure 5 and Video 1). This maneuver enabled the introduction of the SC loop through to the EDWARDS valve deployment device, and by applying tractions, we achieved the correct angulation for crossing the native valve. Ultimately, the SC was retracted to the base of the left ventricle and the Safari 2 guidewire was slightly withdrawn, allowing the SC to be disengaged and removed (Figure 6). Consequently, we were able to deliver the Edwards 26 mm prosthesis, achieving satisfactory device positioning with a grade 1 residual paravalvular leak and a mean transprosthetic gradient measured at 6 mmHg (Figure 7).

**VIDEO 1:** Fluoroscopic images captured during the setup of the Snare catheter (SC). (A) Insertion of the SC via the left femoral artery into the left ventricle. (B) Withdrawal of the SC to the tip of the delivery system. (C) Advancement of the Safari 2 guide through the loop of the SC, enabling further advancement of the SC to the level of the delivery system. Video content can be viewed at https://onlinelibrary.wiley.com/doi/10.1002/ccr3.9628

## 4 Follow‐Up

The next steps of the intervention proceeded without difficulties. The patient was monitored in the cardiac intensive care unit for 24 h post‐procedure and was discharged from the hospital after 48 h, resuming anticoagulant treatment with apixaban. A 6‐month follow‐up revealed no complications related to the approach, and the patient remained asymptomatic.

## 5 Conclusions

This case demonstrated that in complex TAVR procedures characterized by severe aortic stenosis and substantial valve calcification, employing a snare catheter is both technically effective and safe. This strategic approach facilitates the delivery and optimal positioning for the transcatheter heart valve.

## 6 Discussion

In a relatively small number of patients, severe aortic stenosis or an unfavorable calcification pattern of the aortic annulus can hinder the delivery system from crossing the native valve. This situation increases the risk of procedural failure and intra‐procedural complications. Currently, pre‐dilatation during the TAVI procedure is infrequently performed to minimize the potential risk of stroke and massive aortic insufficiency.

According to standard practice, forcing the valve open when it is not possible to advance a valvuloplasty balloon across the valve is not recommended. While deploying the Edwards balloon distally enables the crossing of the valve in some cases, this approach was not feasible in our challenging case.

As a last resort, we utilized the SC to reposition the catheter tip and allow the native valve to be crossed. Previous studies have highlighted the effectiveness of the SC in complex TAVI procedures for device delivery and THV repositioning [1, 2, 3].

For instance, the study of Espinoza Rueda MA et al. reported five cases where the SC was instrumental in guiding the delivery device's positioning and achieving the correct angulation in cases of a horizontal aorta (1). Additionally, the SC has been successfully employed for guiding and deploying an Evolut‐PRO valve during valve‐in‐valve TAVI (2) as well as in situations involving the loss of the delivery device guide (3). Nevertheless, there is a lack of data regarding the failure to cross the aortic valve due to severe stenosis combined with significant calcification. Our case demonstrates that the SC can be utilized to obtain the correct angulation and the stability and traction necessary for the Edwards SAPIENS 3 delivery system to cross the native valve. Traditional techniques, such as pre‐dilation or the use of a rigid guidewire are ineffective in these scenarios.

## Author Contributions

**Simon Berger:** writing – original draft. **Nicolas Combaret:** writing – original draft. **Geraud Souteyrand:** writing – original draft.

## Disclosure

The authors have nothing to report.

## Consent

The authors confirm that written consent for the submission and publication of this case report, including the accompanying images and associated text.

## Data Availability

The data that support the findings of this study are available from the corresponding author upon reasonable request.

## References

[1] M. A. Espinoza Rueda , R. Muratalla González , J. F. García García , et al., “Description of the Step‐By‐Step Technique With Snare Catheter for TAVR in Horizontal Aorta,” JACC Case Reports 3, no. 17 (2021): 1811–1815, 10.1016/j.jaccas.2021.09.006. 34917960 PMC8642723

[2] M. Tsuda , Y. Egami , S. Kawanami , and M. Nishino , “Point‐By‐Point Technique Using a Snare Catheter to Facilitate Self‐Expandable Valve Delivery During Valve‐In‐Valve TAVR,” JACC. Cardiovascular Interventions 15, no. 23 (2022): 2445–2447, 10.1016/j.jcin.2022.09.027. 36480988

[3] M. Tsuda , Y. Egami , S. Kawanami , and M. Nishino , “Simple Bailout Technique Using a Snare Catheter for Wire Withdrawal During Balloon‐Expandable Transcatheter Aortic Valve Implantation: A Case Report,” European Heart Journal ‐ Case Reports 7, no. 9 (2023): ytad417, 10.1093/ehjcr/ytad417. 37662581 PMC10473850

